# A comparative pharmacological study of three Chinese traditional medicines found *Blautia* to be the key functional bacterium of *Coptis chinensis* Franch. and *Phellodendri chinensis* Cortex against colitis

**DOI:** 10.3389/fphar.2025.1587119

**Published:** 2025-06-18

**Authors:** Jiaguo Zhan, Jiale Cheng, Yanan Yang, Xiaofan Xu, Zhiyi Lu, Leilei Li, Haoyu Li, Qiannan Yang, Yanran Hu, Yuxin Song, Qingmiao Fan, Enwei Yang, Qianyue Liang, Shuangyong Sun, Feng Qiu, Ying Cao, Chongming Wu

**Affiliations:** ^1^ School of Chinese Materia Medica, Tianjin University of Traditional Chinese Medicine, Tianjin, China; ^2^ Jinyao Darentang Modern Chinese Medicine Industrial Park Traditional Chinese Medicine Research Institute, Tianjin, China; ^3^ Tianjin Key Laboratory of Therapeutic Substance of Traditional Chinese Medicine, Tianjin, China

**Keywords:** colitis, *Scutellaria baicalensis* Georgi, large intestine dampness-heat syndrome, *Phellodendri chinensis* Cortex, *Coptis chinensis* Franch

## Abstract

**Background:**

Sanhuang refers to the three cold-natured and bitter-flavored traditional Chinese medicines, namely, *Scutellaria baicalensis* Georgi (HuangQin, HQ), *Coptis chinensis* Franch. (HuangLian, HL), and *Phellodendri chinensis* Cortex (HuangBo, HB). Although similar in drug properties, they are traditionally used to treat different dampness-heat syndromes belonging to the Upper Jiao (lung and heart diseases), the Middle Jiao (stomach and intestine diseases), and the Lower Jiao (intestine, kidney, and bladder diseases). The mechanisms behind their differential effects remain unexplored.

**Method:**

A model of large intestine dampness-heat syndrome colitis was established through the administration of exogenous hygrothermal conditions combined with lipopolysaccharide (LPS) and *Escherichia coli*. This model was employed to evaluate the efficacy of *Phellodendri Chinensis* Cortex, *C. chinensis* Franch., and *S. baicalensis* Georgi. Full-length 16S rRNA amplicon sequencing was utilized to assess changes in gut microbiota following drug interventions. Ultimately, the therapeutic effects of key microbial strains on ulcerative colitis were confirmed using a dextran sulfate sodium (DSS)-induced colitis model.

**Results:**

The results showed that HL and HB exhibited significant remedial effects on large intestine dampness-heat syndrome (LIDHS) colitis, but HQ did not. Gut microbial analysis revealed that HL and HB markedly shifted the overall structure of gut microbiota, while HQ showed little impact. The increase of *Blautia* sp. was a common sign in both HL- and HB-treated animals, but it was not observed in the HQ group. On the contrary, the abundance of a *Lactobacillus*-dominant co-abundance gene group (CAG) significantly declined in the HL and HB groups but was similar to the negative control in the HQ group. Additionally, our observations indicate that the enrichment of *Blautia* is consistent with the difference in drug efficacy. *In vivo* experiments also demonstrated the anti-colitis efficacy of *Blautia producta*.

**Conclusion:**

This study identifies *Blautia* as the key bacterium against ulcerative colitis through the establishment of a novel model and a drug comparison.

## 1 Introduction

Ulcerative colitis (UC) is a persistent and relapsing inflammatory disorder characterized by weight loss, abdominal pain, diarrhea, and the potential presence of blood in the stools. The etiology of UC is multifactorial, involving genetic predispositions, environmental influences, alterations in intestinal microecology, and dysregulation of immune function (Chen et al., 2024; Sosna et al., 2023). Epidemiological data indicate that there were more than five million UC patients worldwide in 2023, and the incidence of this disease continues to increase, signifying a global health concern and warrants attention and action from the healthcare community (Yao et al., 2024; Le Berre et al., 2023a). Although there is a growing array of medications for managing UC, their therapeutic efficacy is not satisfactory, and their prolonged usage often leads to various adverse effects, including hepatitis, pancreatitis, hematological abnormalities, and hepatotoxicity (Wei et al., 2022). Furthermore, the duration of remission achieved by UC patients after drug administration typically does not surpass 20%–30% (Le Berre et al., 2023a). New drugs and novel strategies are urgently needed to deal with this health-threatening disease.

Natural products are a rich source of new anti-UC drugs. *Zanthoxylum bungeanum Maxim*, *Houttuyniae Herba*, and *Sanghuangporus* exhibited potent anti-colitis effects *in vitro* and *in vivo* (Qi et al., 2024; Sun et al., 2018; Zhong et al., 2024). As classic traditional Chinese medicines (TCMs), *Phellodendri chinensis* Cortex, *Coptis chinensis* Franch., and *Scutellaria baicalensis* Georgi, interchangeably recognized as “HuangBo” (HB), “HuangLian,” (HL) and “HuangQin” (HQ) in TCM, respectively, and collectively called “Sanhuang,” are historically utilized to treat various diseases caused by damp heat, including large intestine dampness-heat syndrome (LIDHS) colitis (Song et al., 2020; Xie et al., 2022).

It has been well-documented that HQ, HL, and HB can effectively ameliorate colitis in dextran sodium sulfate (DSS)-induced colitis mice (Su et al., 2021; Li et al., 2023). Extracts of HQ and its major metabolites, including baicalin, baicalein, and wogonin, have been extensively documented to ameliorate DSS-induced colitis (Jang et al., 2023). Notably, HQ extracts enhance the relative abundance of beneficial bacteria such as *Lactobacillus* and *Bifidobacterium* while suppressing pathogenic genera like *Clostridium*, *Escherichia*, *Enterococcus*, and *Streptococcus* (Wang et al., 2022). Similarly, extracts and metabolites (e.g., berberine) from HL and HB demonstrate robust anti-colitis effects in DSS models. Berberine specifically upregulates commensal microbes, including *Lactobacillus*, *Roseburia*, *Bacteroides*, and *Akkermansia*, while reducing conditional pathogenic bacteria such as *Alloprevotella* and *Eisenbergiella* (Zhan et al., 2025). This may be attributed to the fact that DSS primarily acts by directly compromising the integrity of the intestinal barrier, which deviates from the physiological mechanism of UC in humans, whereas traditional Chinese medicine theory suggests that they target distinct parts of the human body. Specifically, HQ targets the “upper-Jiao” (from head to diaphragm) and is often prescribed for coughs caused by lung heat. HL addresses the “middle-Jiao” (from diaphragm to umbilicus) botanical drug and addresses ailments like damp-heat jaundice and diarrhea. HB belongs to the “lower-Jiao” (from umbilicus to anus/genitals) and is indicated for conditions such as damp-heat jaundice, urinary challenges, and painful genital afflictions. The intestines belong to the Middle and Lower Jiaos. Due to the distinct targets, HQ, HL, and HB may exhibit different effects in the same disease, including colitis. Consequently, the DSS-induced colitis model does not fully capture the intricate nature of human UC, and special models that align more closely with the symptomatology of UC are needed to evaluate the differences among Sanhuang. Furthermore, clarifying the differences in the therapeutic roles of Sanhuang also helps to discover new drugs for the treatment of UC.

The gut microbiota is a complex and dynamic ecosystem that hosts trillions of microorganisms that play a crucial role in the onset and progression of ulcerative colitis (UC) (Guo et al., 2020). Microbiome studies report low gut microbial richness and diversity in UC patients and a reduced abundance of beneficial bacteria such as *Blautia*, *Bacteroides*, and *A. muciniphila* (Wang et al., 2019; Yu et al., 2019). Meanwhile, accumulated evidence suggests that intestinal dysbiosis may cause dysregulated mucosal immune responses, leading to the onset of IBD in genetically susceptible (Santana et al., 2022). Recent studies have revealed the involvement of mucin-degrading bacterium *A. muciniphila* in the regulation of host barrier function and immune response (Zheng et al., 2022). At the same time, numerous studies have shown that many probiotics and prebiotics can enhance the intestinal barrier to alleviate ulcerative colitis by increasing the colonization of *Lactobacillus*, *Bacteroides*, *A. muciniphila*, and *Blautia* in the gut, as well as the production of beneficial metabolites (Cai et al., 2023; Li et al., 2024; Vesci et al., 2024). The aforementioned evidence indicates that the gut microbiota holds significant potential as a crucial avenue for elucidating the efficacy of botanical drugs and interpreting the TCM theories in relation to UC treatment. Certainly, simultaneously, it behooves us to explore more potentially beneficial bacteria amid the myriad gut microbiota, which may be associated with the anti-ulcerative colitis efficacy of the drug.

In previous studies, dampness-heat colitis was induced using a combination of high-proof liquor (Hongxing Erguotou, 56% ABV), lard (10 g/kg), honey water (30% solution), *Escherichia coli* (intraperitoneal injection), and exposure to a controlled humid-heat environment (Jiang et al., 2025; Yao et al., 2017; Zhang et al., 2024b). In this research, we induced a damp-heat colitis model by subjecting mice to a diet rich in sugar and fat, exposing them to a hot and humid environment, and administering lipopolysaccharide (LPS). We used this model to assess the effects of Sanhuang, namely, HL, HB, and HQ, on UC to discern the differential efficacy of these three botanical drugs. The composition of gut microbiota was also analyzed in detail to provide a potential explanation for the therapeutic effects and the differences among HQ, HL, and HB in the conception of TCM tri-Jiao theory and provide insights for the development of novel anti-colitis medications and potential probiotics.

## 2 Materials and methods

### 2.1 Materials, reagents, and botanical drugs

*Phellodendri chinensis* Cortex (Huang Bo, HB), *C. chinensis* Franch. (Huang Lian, HL), and *S. baicalensis* Georgi (Huang Qin, HQ) were obtained from Anguo Juyaotang Pharmaceutical Co., Ltd. (Hebei, China) and identified according to the requirements of the Chinese Pharmacopoeia (Chinese Pharmacopoeia Commission, 2020). The contents of berberine and phellodendrine in *Phellodendri chinensis* Cortex were 4.4% and 0.41%, respectively; the contents of berberine and coptisine in *C. chinensis* Franch. were 6.5% and 2.0%, respectively; and the content of baicalin in *S. baicalensis* Georgi is 13.8%, which exceeded the quality control level of the Chinese Pharmacopoeia. All three botanical drugs were authenticated by Professor Le-Xin Shu from Tianjin University of Traditional Chinese Medicine using high-performance liquid chromatography (HPLC), and the samples were stored in the School of Chinese Materia Medica, Tianjin University of Traditional Chinese Medicine.

The high-fat/high-sugar (HF/HS) diet D12492 was purchased from Beijing Xiaoshu Youtai Biotechnology Co., Ltd. The mouse enzyme-linked immunosorbent assay (ELISA) kits of TNF-α, IL-1β, and IL-6 were purchased from Jiangsu Meimian Industrial Co., Ltd. (Jiangsu, China). Lipopolysaccharide (LPS) from *Escherichia coli* (*E. coli)* O 111:B4 was purchased from Beijing Solarbio Science and Technology Co., Ltd. (Beijing, China). The human-derived live *Escherichia coli* (nontoxic) strain was provided by Beijing QuantiHealth Technology Co., Ltd. (Beijing, China) (Xu et al., 2023).

### 2.2 Preparation of the aqueous extract of HQ, HL, and HB

Samples (200 g) of *Phellodendri chinensis* Cortex (Huang Bo, HB), *C. chinensis* Franch. (Huang Lian, HL), and *S. baicalensis* Georgi (Huang Qin, HQ) were weighed and then decocted in 1,000 mL of distilled water for 120 min, respectively. After extracting twice, the aqueous extracts were combined, filtered, and then condensed to achieve a solution with a final concentration of 1 g crude botanical drug/mL.

### 2.3 High-performance liquid chromatography (HPLC) analysis

The aqueous extract of HQ (1 g/mL) was diluted to 8 mg/mL with 30% methanol, filtered through a 0.22-μm membrane, and prepared as the test solution. Chromatographic analysis was performed on an Agilent 1260 HPLC system (equipped with a diode array detector (DAD) and a ChemStation workstation, Agilent Technologies, United States) using a Cosmosil C18-M-II column (4.6 mm × 250 mm, 5 μm) at 30°C with a flow rate of 1 mL/min. The injection volume was 10 μL. The mobile phase consisted of methanol (A) and 0.1% phosphoric acid aqueous solution (B) with the following gradient elution program: 0–50 min, 30%–70% A; 50–55 min, 70%–90% A; 55–60 min, 90% A. The detection wavelength was set at 270 nm.

The aqueous extract of HL (1 g/mL) was diluted to 8 mg/mL with the mobile phase (acetonitrile–potassium dihydrogen phosphate solution, 40:60, containing 1.5 g/L sodium dodecyl sulfate), filtered through a 0.22-μm membrane, and prepared as the test solution. Chromatographic analysis was performed on an Agilent 1260 HPLC system (equipped with a DAD detector and a ChemStation workstation, Agilent Technologies, United States) using a Cosmosil C18-M-II column (4.6 mm × 250 mm, 5 μm) at 30°C with a flow rate of 1 mL/min. The injection volume was 10 μL. The mobile phase consisted of acetonitrile and 3.5 g/mL potassium dihydrogen phosphate solution (40:60, with 1.5 g/L SDS). The detection wavelength was set at 345 nm.

The aqueous extract of HB (1 g/mL) was diluted to 4 mg/mL using the initial mobile phase solution, filtered through a 0.22-μm membrane, and prepared as the test solution. Chromatographic analysis was performed on an Agilent 1260 HPLC system (equipped with a DAD detector and a ChemStation workstation, Agilent Technologies, United States) with a Cosmosil C18-M-II column (4.6 mm × 250 mm, 5 μm) at 40°C and a flow rate of 0.8 mL/min. The injection volume was 5 μL. The mobile phase consisted of acetonitrile (A) and water (containing 0.3% phosphoric acid and 0.3% diethylamine, B) with the following gradient elution: 0–10 min, 10%–14% A; 10–20 min, 14%–25% A; 20–45 min, 25% A. The detection wavelength was set at 284 nm.

### 2.4 Large intestine damp heat ulcerative colitis mouse model and TCM treatment

Healthy male Kunming mice (n = 50, body weight: 21–23 g) were purchased from SPF Biotechnology Co., Ltd. (Beijing, China). All experimental procedures were approved by the Animal Care and Use Committee of the Tianjin University of Traditional Chinese Medicine (Authorization number: TCM-LAEC2022). All animals were kept at 22°C–26°C and 55% ± 5% humidity with a 12-h light/dark cycle and allowed free access to food and water during the experiments. After a week of adjustment, the mice were randomly divided into five groups: Normal, Model, HQ, HL, and HB. The Normal group mice were given a regular diet, while the others were fed on a high-fat/high-sugar (HF/HS) diet D12492 for 5 weeks (from week 1 to week 5). Starting from the second week, all animals except the Normal group were put in a high-temperature, high-humidity chamber (temperature 35 °C, 95% humidity) for 8 h every day for a 2-week period (from week 2 to week 4), then were orally administrated with a mixture of lipopolysaccharide (LPS) (2 mg/kg) and live *Escherichia coli* (1 × 10^9^ CFU/animal) for 1 week (on week5) to induce colitis. Afterward, all animals were observed under normal conditions for an additional 2 weeks (from week 6 to week 7). From week 1 to week 7, the HQ, HL, and HB groups were gavaged with HQ (1.5 g raw botanical drug/kg), HL (1.0 g raw botanical drug/kg), or HB (2.0 g raw botanical drug/kg), while the Normal and Model groups were given an equal volume of distilled water. The dosages of HQ, HL, and HB were calculated based on the clinical doses recommended by the Chinese Pharmacopeia (2020 edition). Body weight was recorded every week. At the end of the experiment, all the mice were anesthetized intraperitoneally with 2% pentobarbital sodium (i.p.). Colons were either fixed using 4% paraformaldehyde (Solarbio, China) or cryopreserved at −80 °C. Cecal tissue, inclusive of its contents, was removed for full-length 16S rRNA sequencing. The levels of TNFα, IL-1β, and IL-6 in serum and colonic tissues were measured using the assay kits according to the manufacturer’s instructions (Jiangsu Meimian Industrial Co., Ltd.).

### 2.5 DSS-induced colitis and *Blautia producta* treatment

A total of 24 6-week-old male C57BL/J mice were divided into three groups: Normal group, DSS Model group, and *Blautia producta* (BP) group, with eight mice in each group. Following a 1-week acclimatization period, the BP group received intragastric administration of a quadruple antibiotic regimen for 5 days (vancomycin: 50 mg/kg, neomycin: 200 mg/kg, metronidazole: 200 mg/kg, ampicillin: 200 mg/kg), while the Normal and Model groups were administered distilled water via the same route. After the antibiotic treatment, ulcerative colitis was induced in the mice. During the modeling period, mice in the Model and BP groups had free access to a 3% DSS solution, while those in the Normal group were given distilled water. The BP group was gavage with 10^9^ CFU of bacterial solution (*Blautia producta*) bacterial liquid provided by Beijing Quanti Health Technology Co., Ltd. (Beijing, China). *Blautia producta* administration commenced concurrently with the initiation of DSS exposure and continued daily until the end of the experiment, while the Normal and Model groups received equal volumes of distilled water. Following the initiation of the experiment, the weight of the mice in each group was monitored and recorded starting from the administration period, while the shape of the mice’s feces and hematochezia were noted. At the end of the experiment, all the mice were anesthetized intraperitoneally with 2% pentobarbital sodium (i.p.). Colons were either fixed using 4% paraformaldehyde (Solarbio, China) or cryopreserved at −80 °C (Zou et al., 2024). The levels of TNFα, IL-1β, IL-10, and IL-6 in serum and colonic tissues were measured using the assay kits according to the manufacturer’s instructions (Jiangsu Meimian Industrial Co., Ltd.).

### 2.6 Disease activity index (DAI) assessment

The mice were checked daily for body weight, stool consistency, and the presence of gross blood in feces and at the anus. The DAI was calculated according to the previous scheme (Sun et al., 2018). The DAI was computed as: DAI = (body weight loss score + stool consistency score + hematochezia score)/3.

### 2.7 Histology of colon

Colonic tissues were washed in phosphate-buffered saline (PBS), and then the colonic specimens were fixed in 10% paraformaldehyde (Solarbio, China). After embedded in paraffin, the colon sections were deparaffinized with xylene, rehydrated, and stained with hematoxylin and eosin (H&E). Histological damage was evaluated using a validated scoring system as described previously (Zou et al., 2024). Lesions were given semi-quantitative scores ranging between 0 and 4, based on the severity of the lesion. The scale was as follows: normal (0), mild (1), moderate (2), severe (3), and very severe (4).

### 2.8 Full-length 16S rRNA sequencing and gut microbiota analysis

The cecal DNA was extracted as previously reported (Li et al., 2018). The V1-V9 region of the bacteria 16S ribosomal RNA gene was amplified by PCR. The recovery of amplicons, the construction of SMRTbell libraries, and the sequencing on an Illumina HiSeq 2500 instrument were performed by Shanghai Biozeron Biotechnology Co. Ltd. (Shanghai, China) as described by Yang et al. (Yanan et al., 2023). The resulting FASTA sequences were subjected to quality control and alignment processes. The alpha diversity and beta diversity of the gut microbiome were analyzed by the vegan package (v2.7) of R version 4.0.2. The difference between the Bray–Curtis distance of Principal coordinate analysis (PCoA) and clustering analysis at the operational taxonomic unit (OTU) level was assessed by Adonis analysis. Correlation analysis was calculated by the Spearman algorithm.

### 2.9 Statistical analysis of data

The pharmacological data are expressed as the mean ± SEM and analyzed using Prism 7 (GraphPad). One-way ANOVA and Dunnett’s test were used to assess the differences among groups. *P* < 0.05 was considered statistically significant.

## 3 Result

### 3.1 Identification of botanical drugs and their metabolites

**FIGURE 1:**
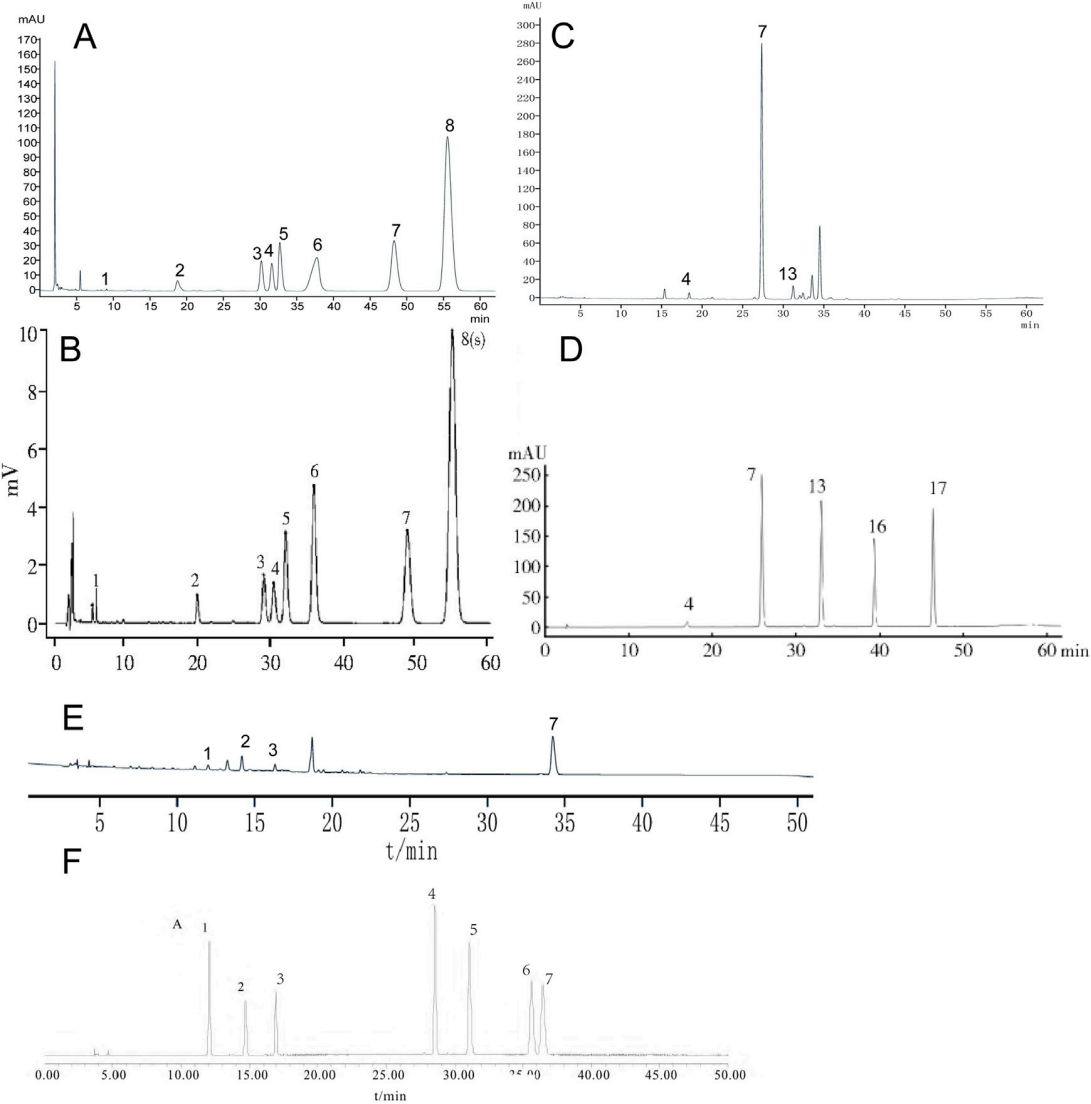
The chemical profile of HL, HQ, and HB by HPLC analysis. **(A)** HL test sample, **(B)** The comparative characteristics map of HL: 1. magnoflorine, 3: columbamine, 4. jatrorrhizine hydrochloride, 5. epiberberine, 6. coptisine, 7. palmatine chloride, 8. *Berberini hydrochloridum*. **(C)** HQ test sample. **(D)** The comparative characteristics map of HQ: 4. scutellarin, 7. baicalin, 13. wogonoside, 16. baicalein, 17. wogonin. **(E)** HB test sample. **(F)** The comparative characteristic map of HB: 1. chlorogenic acid, 2. phellodendrine chloride, 3. magnoflorine, 4. jatrorrhizine hydrochloride, 5. berberrubine, 6. palmatine hydrochloride, 7. *Berberini hydrochloridum*.

To identify the three botanical drugs and their metabolites, we employed high-performance liquid chromatography (HPLC). HPLC analysis revealed that the aqueous extract of HL was rich in magnoflorine, columbamine, jatrorrhizine hydrochloride, epiberberine, coptisine, palmatine chloride, and berberine hydrochloride. The extract of HQ predominantly contained scutellarin, baicalin, and wogonoside, while HB predominantly contained chlorogenic acid, phellodendrine chloride, magnoflorine, and berberine hydrochloride (Figure 1).

### 3.2 Establishment of LIDHS colitis

**FIGURE 2:**
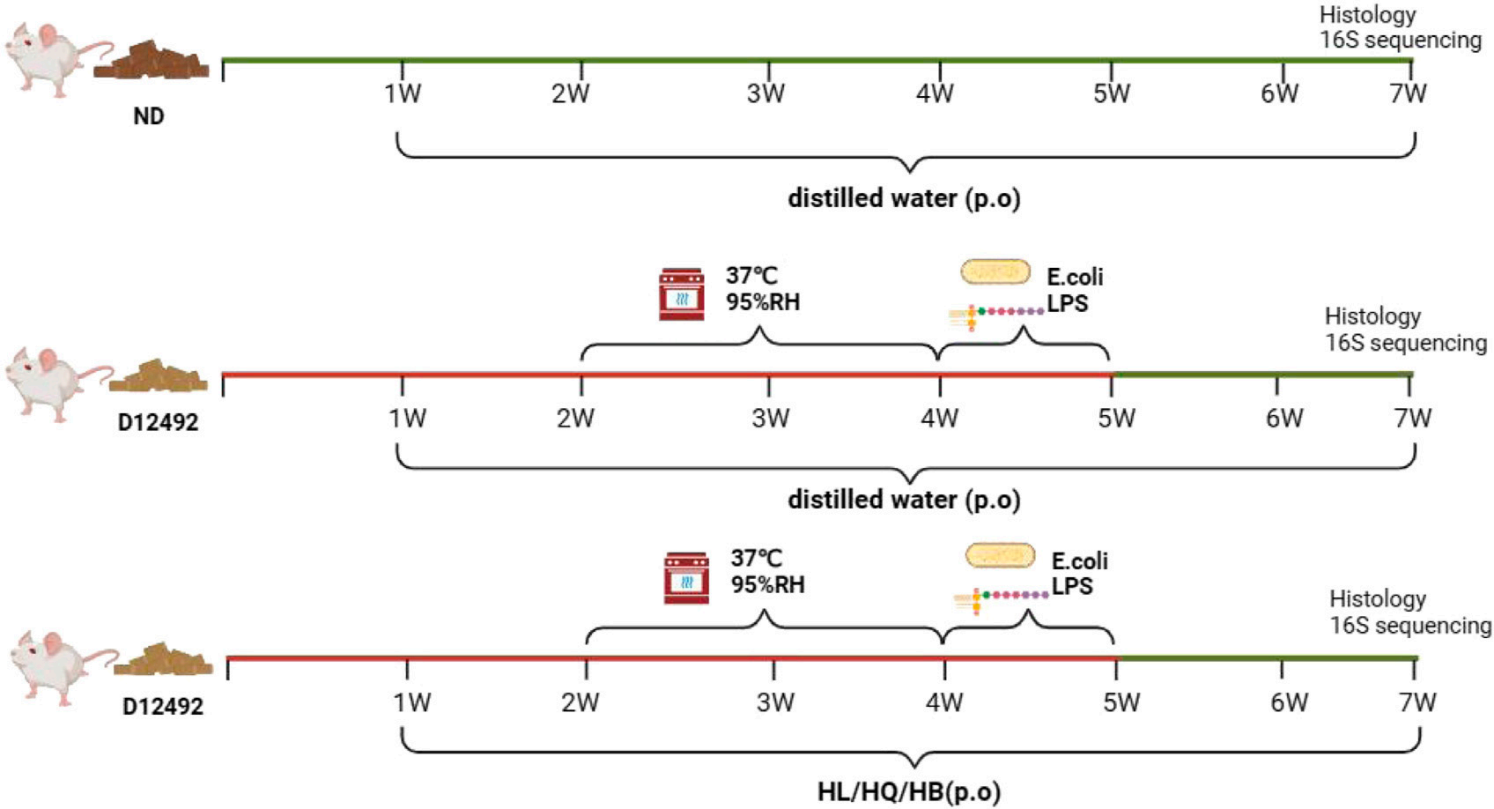
Establishment of large intestine dampness-heat syndrome (LIDHS) colitis.

**FIGURE 3:**
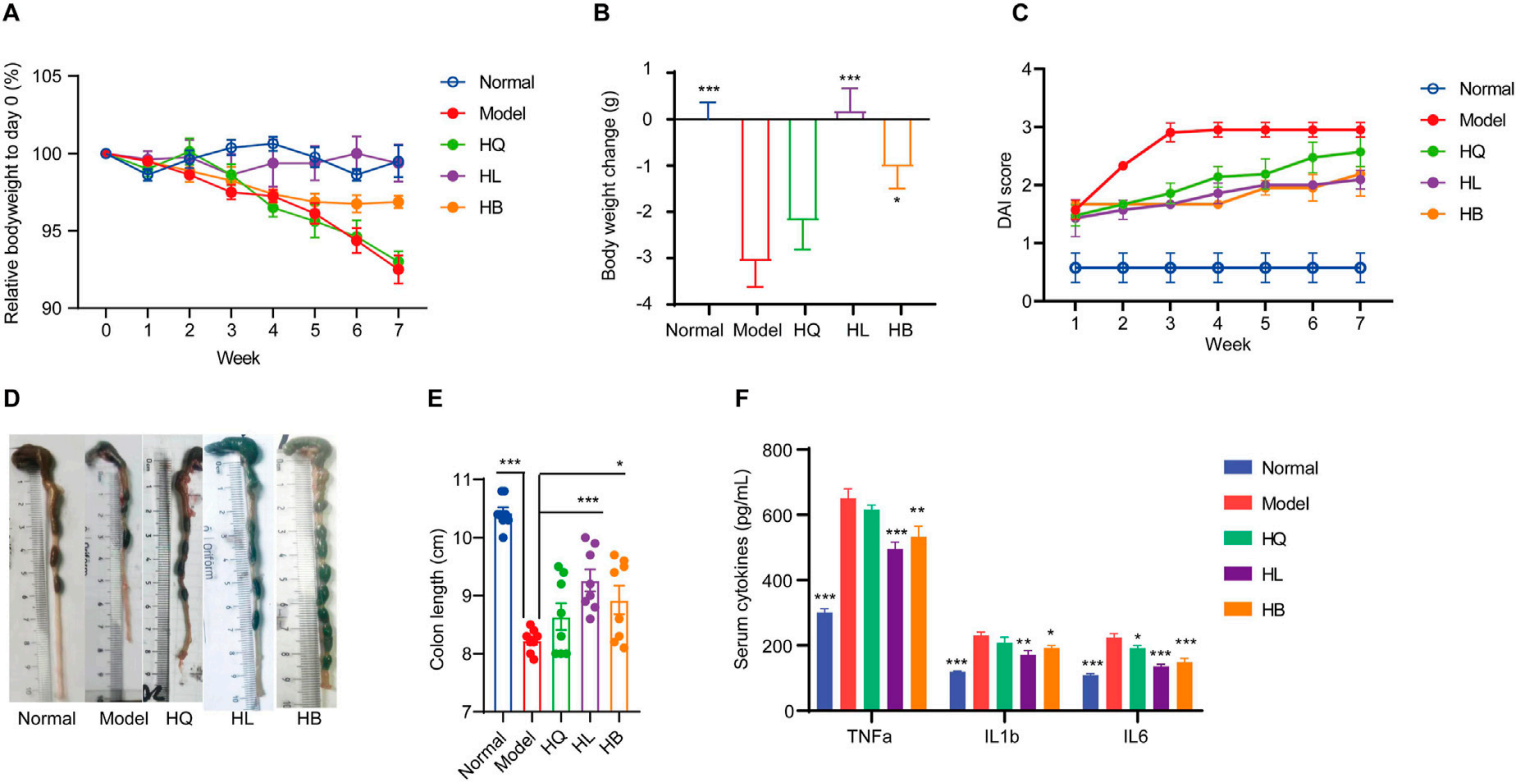
The effect of Sanhuang (HL, HB, and HQ) on general physiological indices in LIDHS colitis mice. **(A)** The relative body weight on day 0 (%). **(B)** Body weight change (g). **(C)** DAI score of each group. **(D)** Representative photographs of the colon tissue. **(E)** The colon length was measured with ImageJ software. **(F)** Serum levels of TNF-α, IL-1β, and IL-6. N = 8. *P < 0.05, **P < 0.01, and ***P < 0.001.

A LIDHS colitis model was created by a combination of a high-sugar and high-fat diet, high-temperature, high-humidity cultivation, and a live *Escherichia coli* (nontoxic) and lipopolysaccharide (LPS) trigger (Figure 2). The Normal group displayed healthy behavior, such as active and spirited mice with smooth, white, clean, and shiny fur, regular appetite, stools and urine, and steady weight gain. In contrast, the LIDHS model animals exhibited typical signs of LIDHS, including increased water consumption, more frequent urination with a yellowish tint, and initially soft, yellow stools. Prolonged exposure to a high-temperature and high-humidity environment led to reduced, darker urine and initially sticky, yellow stools, transitioning to looser stools in some mice. During their time in the hot chamber, these mice appeared restless, with reduced appetite, dull fur, and noticeably drooping scrotums. However, upon removal from the chamber, their demeanor improved gradually, though weight loss was evident. When exposed to LPS and live *Escherichia coli*, the LIDHS mice exhibited symptoms of distress: reduced activity, loss of appetite, chilling, light sensitivity, huddling together, eyelid swelling, and increased secretions. After transfer to the normal condition, their symptoms became worse, marked by severe lethargy, diarrhea, tangled and dull fur, inflammation around the anus, and the release of loose, foul-smelling, sometimes bloody stools. Simultaneously, the animals also displayed colitis symptoms, including elevated DAI index, diarrhea, and systemic inflammation (Figure 3). These results indicate that modulation of dietary and environmental factors can replicate the syndrome of LIDHS colitis, resulting in a suitable model that is closer to the causes and phenotypes of UC.

### 3.3 HL and HB but not HQ attenuate symptoms in LIDHS colitis mice

**FIGURE 4:**
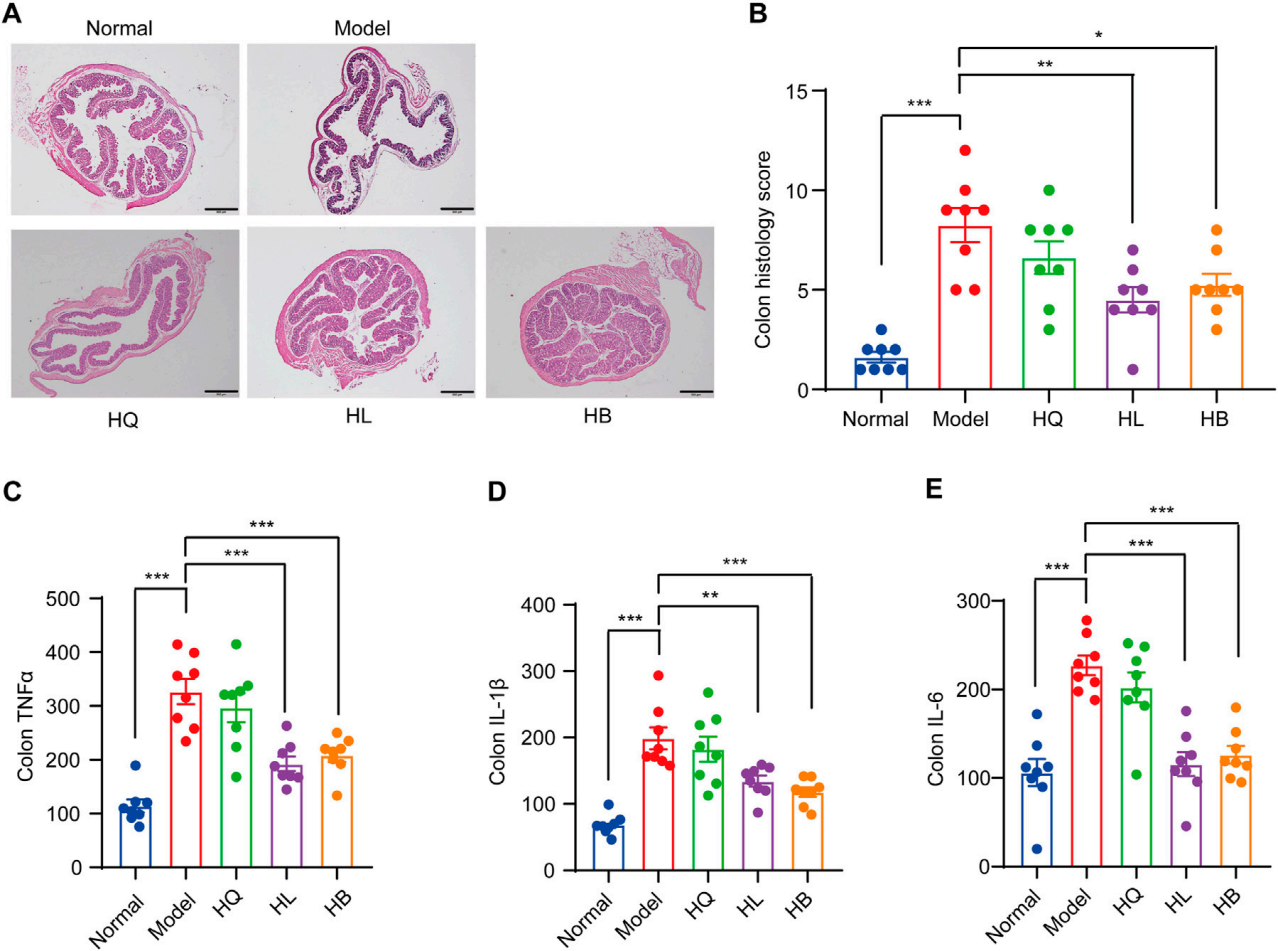
The effect of Sanhuang (HL, HB, and HQ) on colonic damage in LIDHS colitis mice. **(A)** Representative pictures of H&E staining for colon tissues among each group. **(B)** Histopathological scores were analyzed from slides. The colonic levels of **(C)** TNF-α, **(D)** IL-1β, and **(E)** IL-6. N = 8. *P < 0.05, **P < 0.01, and ***P < 0.001.

First, the general symptoms of LIDHS colitis to assess the effects of HL, HB, and HQ were observed. The HL and HB groups showed similar food intake and activity with the Normal group. Their weight loss was notably less than that of the Model group (Figures 3A, B). Consequently, the DAI scores decreased while colon length increased (Figures 3C–E). In addition, the serum levels of pro-inflammatory cytokines TNF-α, IL-1β, and IL-6 were significantly decreased in the HL and HB groups compared to the negative control group (Figure 3F). However, these improvements were not observed in HQ-treated animals. (Figure 3). Although it has been reported that HQ is also effective in alleviating colitis in DSS-induced models (Cui et al., 2021), HQ showed little effect on LIDHS colitis in terms of body weight, DAI score, colon length, and inflammatory cytokine levels (Figure 3). We further evaluated the impact of HQ, HL, and HB on the histology and inflammation status of colon tissue. Compared to the Normal group, the Model group exhibited pronounced colon damage such as mucosal erosion, hyperemia, cryptal gland loss, goblet cell depletion, and an influx of inflammatory cells at the lesion sites (Figures 4A, B). In accordance, the colonic levels of inflammatory cytokines TNF-α, IL-1β, and IL-6 were significantly higher in the Model group than in the Normal group (Figures 4C–E). Treatment with HL and HB substantially rectified these pathological manifestations (Figures 4A–E), which underscores the effectiveness of HL and HB to protect the colon against damp, heat, and pathogen invasion. Again, the efficacy of HQ was quite slight and remarkably weaker than HL and HB (Figure 4).

The deviation of HQ from HL and HB in the treatment of LIDHS colitis contradicts the observation in the chemical-induced colitis model. However, it is in line with the TCM tri-Jiao theory, as HL and HB target the Middle and Lower Jiaos, which enclose the site of the intestine, while HQ belongs to the Upper Jiao, which corresponds to the heart and lung but not the intestine. These findings suggest that LIDHS colitis is a suitable model to evaluate the therapeutic effect of traditional Chinese medicines on colitis accompanied by damp-heat symptoms.

### 3.4 HL and HB but not HQ significantly modulate gut microbiota in LIDHS colitis mice

**FIGURE 5:**
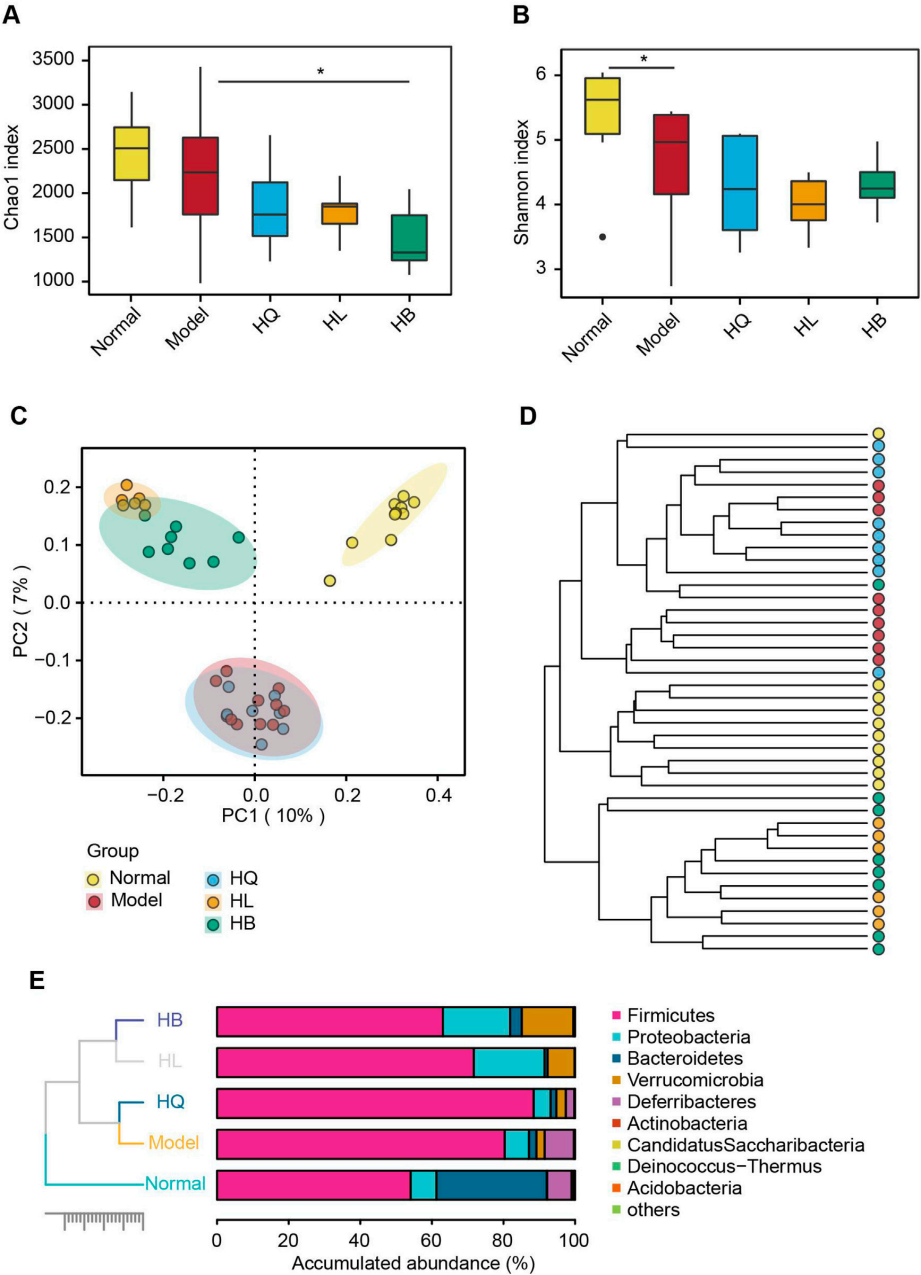
The effect of Sanhuang (HL, HB, and HQ) on gut microbial diversity in LIDHS colitis mice. The alpha diversity is assessed by the **(A)** Chao1 and **(B)** Shannon indices. **(C)** Principal coordinate analysis (PCoA) based on the Bray–Curtis distance. **(D)** Hierarchical clustering analysis based on the Bray–Curtis distance at the operational taxonomic unit (OTU) level. **(E)** Taxonomic profile of the gut microbiota at the phylum level. N = 8. *P < 0.05.

Gut microbiota analysis revealed that the LIDHS colitis mice showed declined alpha diversity, especially the Shannon index, compared to mice in the Normal group. Treatment with Sanhuang further decreased the alpha diversity of gut microbiota, which is in accordance with their antibacterial activities (Figures 5A, B). Principal coordinate analysis (PCoA) displayed a distinct separation of the gut microbiota of the LIDHS colitis group from the Normal group. Intriguingly, treatment with HL and HB significantly shifted the gut microbial structure from the Model group, while the HQ group showed little shift but substantially overlapped with the Model group (Figure 5C). Hierarchical clustering at the OTU and phylum levels also revealed that HL and HB groups clustered together and departed from the Model group, while the HQ group aligned more closely with the Model group (Figures 5D, E). These changes in gut microbiota are in harmony with the pharmacological outcomes; that is, the therapeutic efficacy of HQ, HL, and HB on LIDHS colitis is relevant to their modulation of gut microbiota.

### 3.5 Key bacteria that are closely related to the effectiveness of Sanhuang on LIDHS colitis mice

**FIGURE 6:**
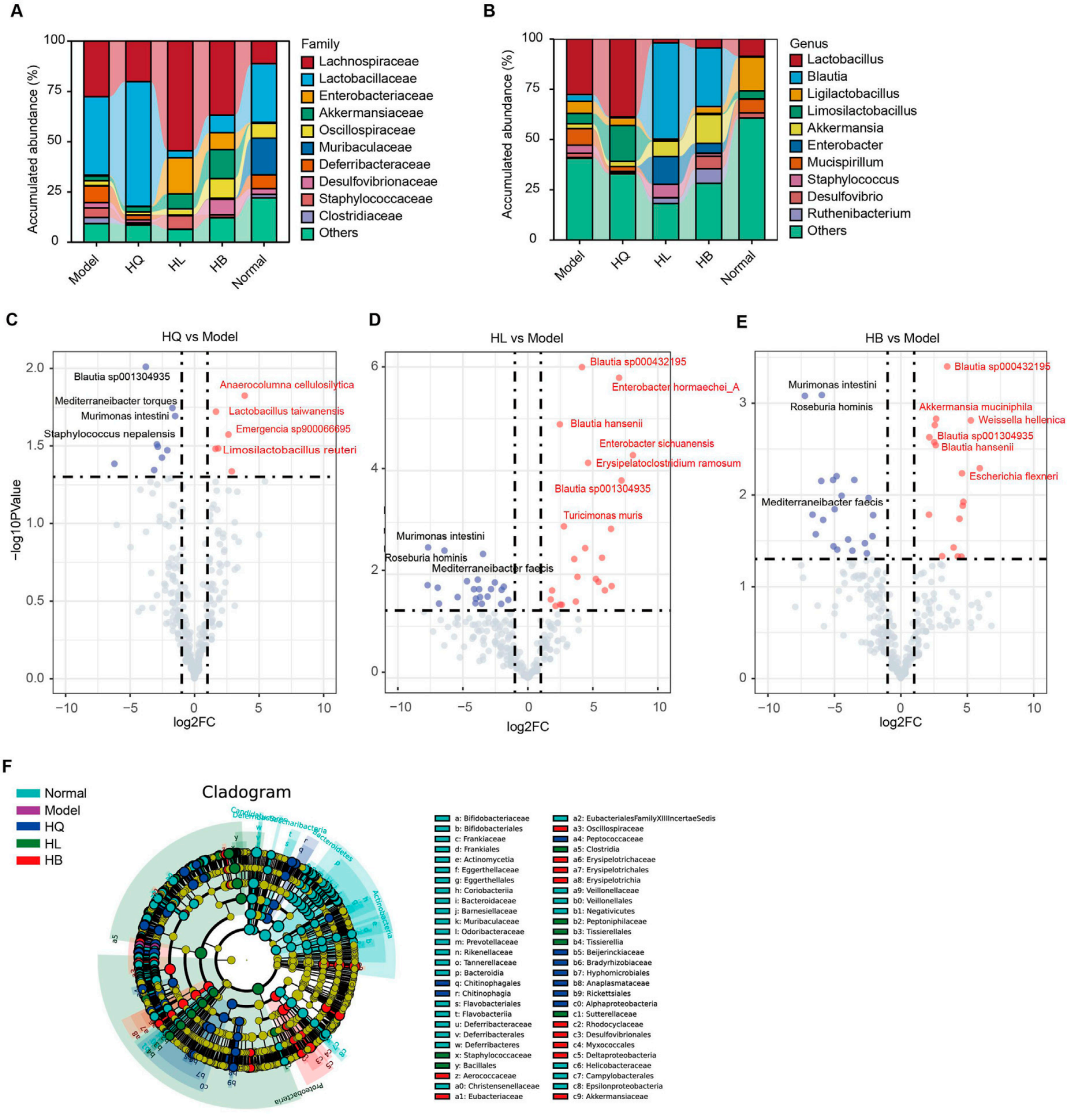
The effect of Sanhuang (HL, HB, and HQ) on gut microbial composition in LIDHS colitis mice. Taxonomic profile of the gut microbiota at the **(A)** family and **(B)** genus levels. **(C)** The volcano plot highlights the differential gut microbial species between the Model and the HQ groups. **(D)** The volcano plot highlights the differential gut microbial species between the Model and the HL groups. **(E)** The volcano plot highlights the differential gut microbial species between the Model and the HB groups. **(F)** Linear discriminant analysis effect size (LEfSe) analysis comparing the differential microbiota among the groups. N = 8.

As the gut microbiota is closely related to the efficacy of HQ, HL, and HB on LIDHS colitis, we compared the difference in the gut microbial composition among groups in detail in order to find key bacteria that may mediate the anti-colitis effect of HL and HB. Compared to the LIDHS colitis mice, the most striking change in the gut microbial community of the HL and HB groups is the increase of *Blautia*, *Akkermansia, and Enterobacter*, and the decrease of *Lactobacillus* and *Limosilactobacillus* (Figures 6A, B). In contrast, the HQ group did not show a similar increase of *Blautia*, *Akkermansia, and Enterobacter* to the HL and HB groups but did enlarge the abundance of *Lactobacillus* and *Limosilactobacillus* (Figures 6A, B).

Volcano analysis with a stricter criterion (|fold-change (FC)| > 2, p-value <0.05) highlighted several species with marked variances among groups. HQ treatment boosted six species, including *Anaerocolumna cellulosilytica*, *Lactobacillus taiwanensis*, *Limosilactobacillus reuteri*, and *Emergencia sp900066695*, but reduced nine species, including *Blautia sp001304935*, *Mediterraneibacter torques*, *Murimonas intestini*, and *Staphylococcus nepalensis* (Figure 6C). *Lactobacillus* has been extensively reported to ameliorate DSS-induced colitis. In this study, compared to LIDHS colitis mice, HQ significantly enriched the abundance of *Lactobacillus* (Figures 6A, B) yet failed to exhibit a therapeutic effect. This suggests that the *Lactobacillus* enriched by HQ in dampness-heat type colitis may exhibit strain-specific characteristics (e.g., lacking anti-inflammatory or gut barrier-repairing properties) (Li et al., 2023). In contrast, HL and HB changed more species than HQ. Notably, *Blautia* sp. showed a consistent increase following HL and HB treatment, while *Murimonas intestini*, *Roseburia hominis*, and *Mediterraneibacter faecis* constantly decreased across HL and HB groups (Figures 6D, E). Moreover, HL administration expanded the abundance of *Enterobacter hormaechei_A*, *Enterobacter sichuanensis*, *Erysipelatoclostridium ramosum*, and *Turicimonas muris* (Figure 6D). HB treatment amplified species like *Akkermansia muciniphila*, *Weissella hellenica*, and *Escherichia flexneri* (Figure 6E). Linear discriminant analysis effect size (LEfSe) analysis also found that HL marked enriched *Staphylococcaceae, Bacillales*, and Clostridia; HL enriched Eubacteriaceae, Erysipelotrichaceae, *Akkermansia muciniphila*, and *Escherichia flexneri;* and HQ enriched Peptococcaceae, Beijerinckiaceae, Bradyrhizobiaceae and Anaplasmataceae (Figure 6F). Overall, the enrichment of *Akkermansia* and *Blautia* is closely related to the anti-colitis effect of HL and HB. These two genera may play a pivotal role in the therapeutic efficacy of HL and HB against LIDHS colitis.

### 3.6 Co-abundance gene groups (CAGs) of gut microbiota following Sanhuang (HL, HB, and HQ) treatment in LIDHS colitis mice

**FIGURE 7:**
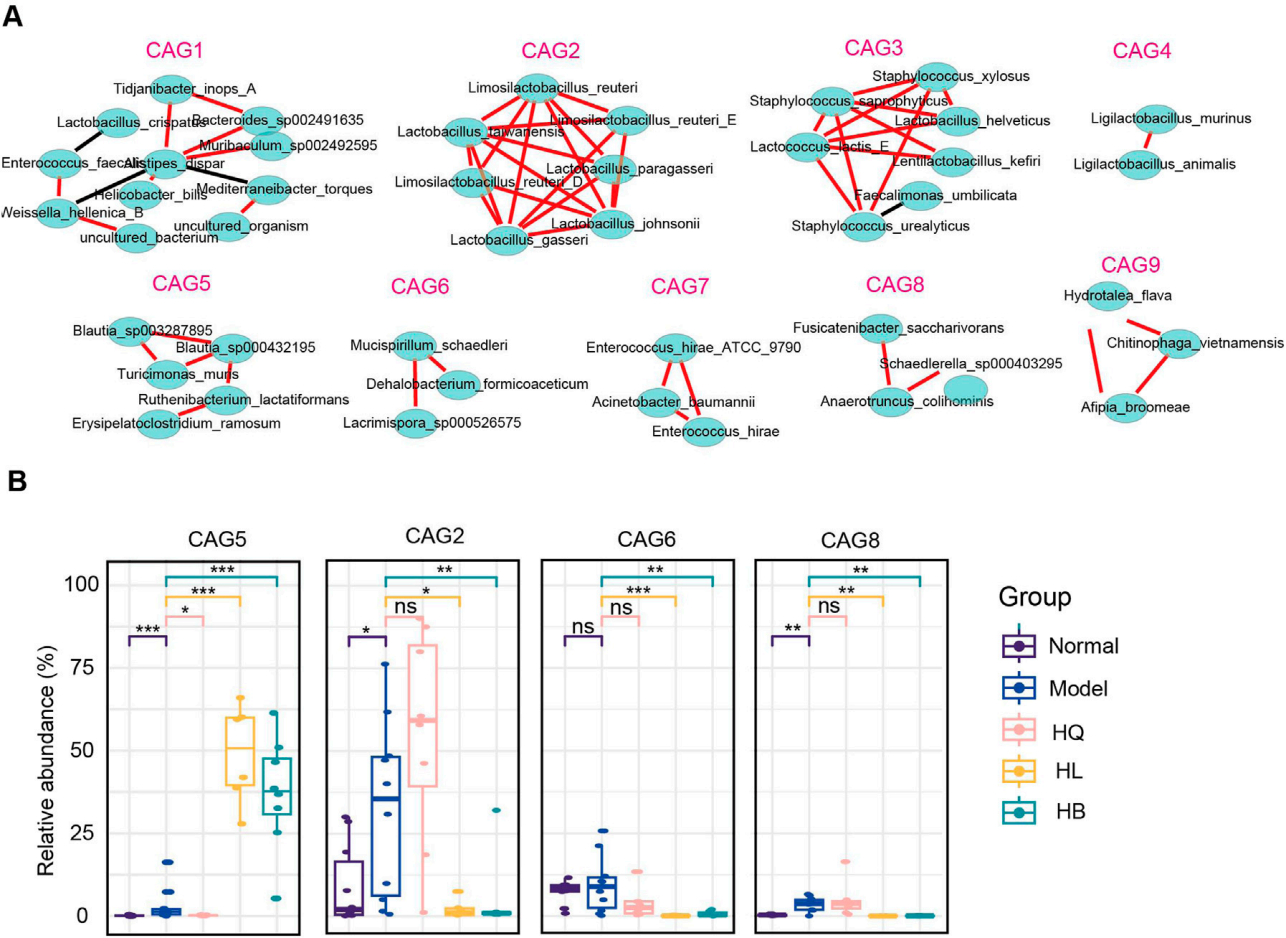
The functional groups of the gut microbiota following Sanhuang (HL, HB, and HQ) treatment. **(A)** SparCC analysis to identify co-abundance gene groups (CAGs) of gut microbiota following Sanhuang (HL, HB, and HQ) treatment. **(B)** The relative abundance of differential CAGs (CAG5, CAG2, CAG6, CAG8) among five groups. N = 8. *P < 0.05, **P < 0.01, and ***P < 0.001.

Co-abundance gene groups (CAGs) help to identify the bacteria that change in harmony and may exert a similar function (Yang et al., 2022a). A total of nine CAGs were constructed by SparCC analysis with a correlation coefficient >0.4 and a p-value <0.05 (Figure 7A). CAG1 contained 11 bacterial species: *Tidjanibacter inops_A*, *Bacteroides sp002491635*, *Lactobacillus crispatus*, *Muribaculum sp002492595*, *Mediterraneibacter torques*, *Enterococcus faecalis*, *Weissella helenica_B*, *Helicobacter_bilis* and so on. CAG2 and CAG4 predominantly comprised lactic acid bacteria: *Limosilactobacillus*, *Lactobacillus*, and *Ligilactobacillus*. CAG3 featured *Staphylococcus* species (*S. xylosus*, *S. saprophyticus*, *S. urealyticus*), and lactic acid bacteria like *Lactococcus lactis_E*, and *Lactobacillus helveticus*. CAG5 integrated short-chain fatty acid (SCFA)-producing bacteria, like *Blautia* species (*B. sp003287895* and *B. sp000432195*), with lactic acid-producers, such as *Ruthenibacterium lactatiformans* and other species, including *Turicimonas muris* and *Erysipelatoclostridium ramosum*. CAG6 also included SCFA-producing bacteria like *Mucispirillum schaedleri* and *Lacrimispora sp000526575*, along with a dichloromethane-degrading bacteria *Dehalobacterium formicoaceticum*. CAG7 primarily consisted of pathogens, notably *Enterococcus hirae_ATCC_9790*, *Acinetobacter baumannii*, and *Enterococcus hirae*. CAG8 embraced butyrate producer *Anaerotruncus colihominis*, SCFA-producing bacteria *Fusicatenibacter saccharivorans*, and *Schaedlerella sp000403295*. CAG9 contained species like *Hydrotalea flava*, *Chitinophaga vietnamensis,* and *Afipia broomeae* (Figure 7A).

The most marked difference between the effective botanical drugs HL and HB and the ineffective botanical drug HQ was that HL and HB enriched CAG5 but declined CAG2, CAG6, and CAG8, while HQ showed much less or no impact on them (Figure 7B). As CAG5 is dominant in *Blautia* sp., these butyrate-producing bacteria might play a crucial role in the anti-colitis effect of HL and HB. Although it has been broadly reported that *Lactobacillus* sp. is effective in ameliorating DSS-induced colitis, the increase of *Lactobacillus* sp. by HQ contributed little to the anti-colitis effect.

### 3.7 HL/HB-enriched *Blautia* alleviates DSS-induced colitis in mice

Numerous studies have reported that *Akkermansia* can enhance the management of ulcerative colitis by modulating the host’s immune response and promoting the production of beneficial metabolites. However, there is a paucity of research on the anti-colitis effects of *Blautia*. Recently, more species-targeting research on *Blautia* has been released. Decreased *Blautia luti* was detected in the gut ecosystem of obese children and related to the worsening of intestinal inflammation and metabolic phenotype (Benítez-Páez et al., 2020). Our previous research has demonstrated that *Bl. producta* exhibits a significant anti-hyperlipidemic effect, frequently associated with chronic inflammation. Prior studies have highlighted its crucial role in mediating the anti-inflammatory response in HT-29 intestinal epithelial cells (Pathmanathan et al., 2020). Consequently, we sought to further investigate the protective effects of *Bl. producta* in the context of ulcerative colitis.

**FIGURE 8:**
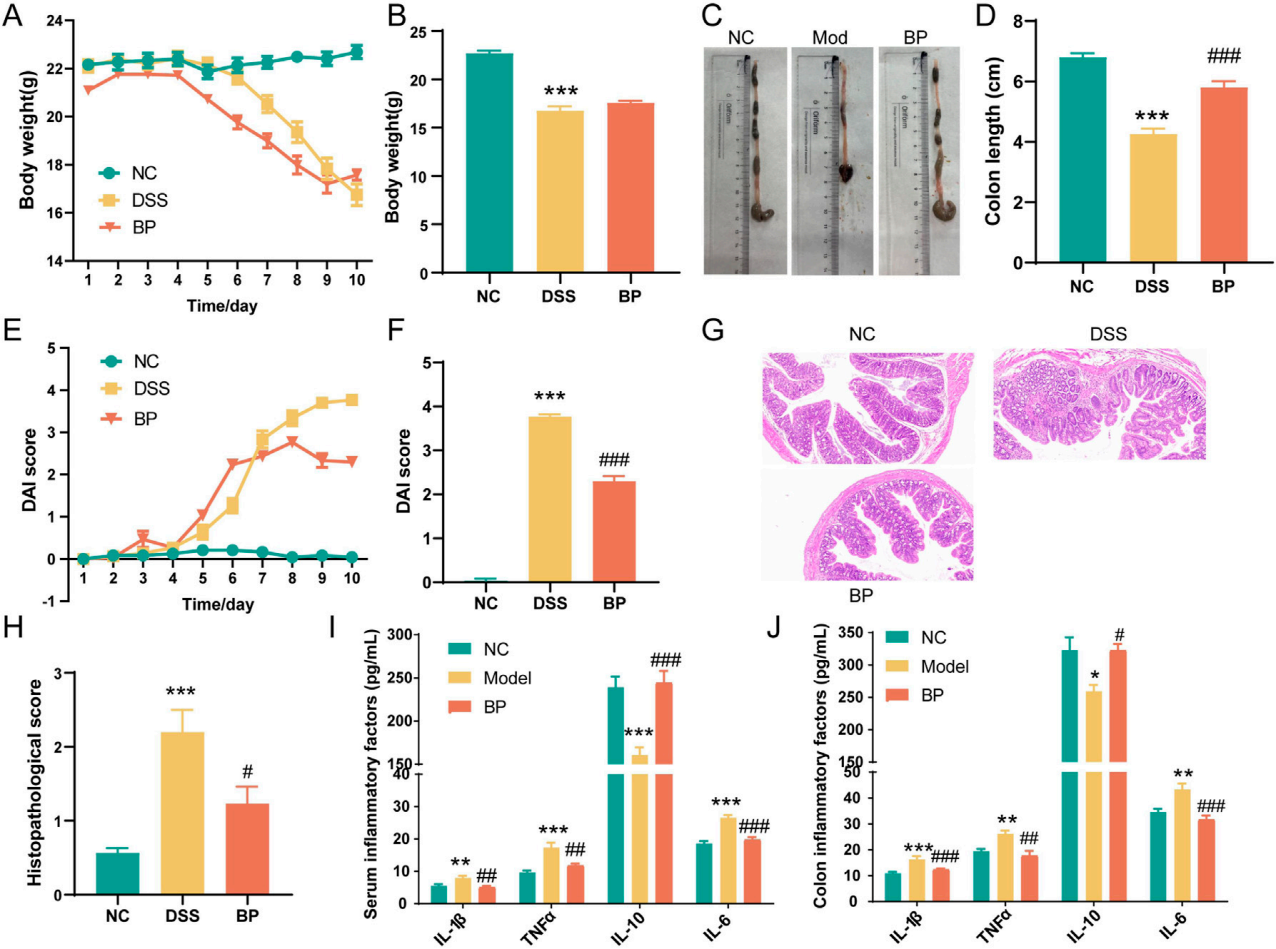
*Bl. producta* alleviates DSS-induced colitis in mice. **(A)** Body weight curve. **(B)** Body weight on the 10th day. **(C, D)** Colon length. **(E)** DAI score curve. **(F)** DAI score on the 9th day. **(G)** H&E staining of colon tissue. **(H)** Histological scores of the colons of the different groups. **(I)** Concentrations of serum inflammatory cytokines. **(J)** Concentrations of colon inflammatory cytokines. N = 8 for each group. *P < 0.05, **P < 0.01, and ***P < 0.001.

Experimental results showed that the weight of mice in the control group continued to increase, as seen in Figures 8A, B. However, after 6 days of modeling, mice given DSS experienced a significant decrease in weight, along with severe diarrhea and bloody stools. The weight loss caused by DSS was alleviated 9 days after *Bl. producta* administration. Visual observation revealed that the colon in the DSS group became severely congested and dark in color. In contrast, after *Bl. producta* administration, the colon appearance of mice with UC returned to normal, accompanied by a reduction in the DAI score (Figures 8C–F). Histological images demonstrated that the colon base in the DSS group became thinner, with broken epithelial cells, reduced crypts and goblet cells, and a compromised colon barrier. As anticipated, treatment with *Bl. producta* significantly alleviated these symptoms and reduced the colon histopathology score (Figures 8G, H). Given that intestinal and systemic inflammation are common in UC patients, we also investigated the effects of *Bl. producta* on both colonic and systemic inflammation in the DSS Model mice. As shown in Figures 8I, J, the administration of *Bl. producta* significantly inhibited the levels of colon and serum inflammatory factors TNF-α, IL-6, and IL-1β and increased the levels of the anti-inflammatory factor IL-10. These experimental results consistently indicate that HL/HB-enriched *Bl. producta* can effectively improve experimental colitis.

## 4 Discussion

Nowadays, improper diet due to the accelerated pace of work and life, changes in diet structure (indulgence in a richly fatty, sweet, and full-flavored diet and drinking), and emotional stress and depression heavily disrupt the integrity of the intestinal barrier and our immune systems and further induce UC. Although chemical-induced colitis models have been successfully used to discover effective anti-UC drugs, their pathogenic mechanisms are not the same as the general pathogenesis of UC. Herein, we established a LIDHS colitis model that is more in line with the development of UC with LIDHS syndrome in humans. This syndrome is induced by exogenous dampness-heat and inactivated *Escherichia coli*, making it more representative of the development of UC and more appropriate for evaluating the efficacy of various traditional natural medicines. We then used this model to assess the pharmacological effect of three popular traditional Chinese botanical drugs with known anti-colitis activity. We found that *Phellodendri chinensis* Cortex (HB) and *C. chinensis* Franch. (HL) were effective in alleviating LIDHS colitis, while *S. baicalensis* Georgi (HQ) was not. These results somewhat contradict previous reports but are in accordance with the TCM tri-Jiao theory and clinical practice. Further investigation found that gut microbiota, especially the increase of *Blautia* and *Akkermansia*, mediate the anti-UC effect of HL and HB.

Within the theoretical framework of TCM, the human body can be divided into the Upper Jiao (Shang Jiao), the Middle Jiao (Zhong Jiao), and the Lower Jiao (Xia Jiao). The Upper Jiao encompasses the heart, lungs, head, and face and functions similarly to a mister that supports the other parts of the body. The Middle Jiao, including the spleen, stomach, liver, and gallbladder, plays a role in digesting food, absorbing, and distributing the essence of food to produce Qi and blood. The Lower Jiao, comprising the small intestine, large intestine, kidney, and urinary bladder, centers on the expulsion of waste and urine (Li et al., 2014). HQ, HL, and HB, collectively called Sanhuang, are known for their properties of clearing dampness and dispelling heat. Among them, HQ is traditionally prescribed to address Upper Jiao ailments resulting from dampness-heat; HL is believed to clear dampness-heat in the Middle Jiao; HB is customarily used to counteract Lower Jiao diseases caused by dampness-heat. All three botanical drugs have been documented to possess anti-colitis effects (Li et al., 2022a; Zhang et al., 2024a; Zhang et al., 2022). However, they should exhibit differential efficacy according to TCM tri-Jiao theory, as they target different parts of our body. To assess the tri-Jiao theory, we tested the pharmacological effects of HQ, HL, and HB on the TCM-related LIDHS colitis model. Our results confirmed the anti-colitis effect of HB and HL. However, HQ showed minimal effectiveness on LIDHS colitis. The distinct effects of HQ, HL, and HB on LIDHS colitis might be attributed to the fact that LIDHS primarily originates in the gastrointestinal tract, rendering the effect of HQ (an Upper Jiao-targeting botanical drug) relatively mild. These findings strongly suggest that the distinct action sites within the three Jiaos of these botanical drugs significantly influence their protective outcomes against LIDHS colitis, offering fresh experimental support for the tri-Jiao theory of TCM.

It has been well-documented that gut microbiota mediates the anti-colitis effects of many drugs, and multiple bacteria are effective in migrating colitis (Wang et al., 2024). However, the special bacteria that mediate the UC-ameliorating effects of HL and HB remain largely unknown. In our study, we observed an increase in *Lactobacillus* and *Staphylococcus* but a decrease in *Ligilactobacillus* in the LIDHS colitis model. Treatment with HB and HL led to a reduced relative abundance of *Lactobacillus* but notably boosted the anti-inflammatory and butyrate-producing bacteria, especially *Blautia* (*B. sp000432195*, *B. sp001304935*, and *B. hansenii*) and *Akkermansia muciniphila*. Compelling evidence proves that IBD patients are usually found to have a decrease in *Akkermansia muciniphila* and *Blautia* (Bolte et al., 2021; Earley et al., 2019), whereas supplementation of *Akkermansia muciniphila* and *Blautia* stunningly attenuates DSS-induced colitis by restoring the balance of gut microbiota and intestinal metabolism (Wang et al., 2023; Wu et al., 2023; Xue et al., 2023). Furthermore, a clinical study also found that the abundance of *Blautia* was significantly increased in patients who achieved clinical remission after fecal microbiota transplantation (FMT) (Schierová et al., 2020). Hence, it is reasonable to infer that these two kinds of microbes might be key functional bacteria that mediate the anti-LIDHS colitis effect of HL and HB. It is also widely reported that *Lactobacillus* sp. are beneficial bacteria to migrate colitis in a DSS-induced model. However, HQ significantly increased the abundance of *Lactobacillus* sp. but showed no effectiveness. This suggests that the *Lactobacillus* enriched by HQ in dampness-heat type colitis may exhibit strain-specific characteristics (e.g., lacking anti-inflammatory or gut barrier-repairing properties).

Recently, an increasing number of studies have focused on the species within the genus *Blautia*. *Bl. producta* is a significant anaerobic bacterium derived from the human gut microbiota, and recent research has uncovered its various potential roles in health and disease. Our previous studies demonstrated that *Bl. producta* can effectively ameliorate hyperlipidemia by promoting the production of the beneficial metabolite 12-methyl myristic acid (12-MMA) (Xu et al., 2023). Additionally, another study suggested that *Bl. producta* may enhance the expression of LDL receptor (LDLR) in the liver, thereby augmenting the cholesterol-lowering effects of berberine (Yang et al., 2022b). A cross-sectional study involving Italian adults revealed that the beneficial effects of probiotics on intestinal health are closely associated with the abundance of *Bl. producta* (Toscano et al., 2017). However, only a limited number of studies have examined the role of *Bl. producta* in gastrointestinal diseases and inflammation. Clinical investigations have shown that the abundance of *Bl. producta* in the gut microbiota of patients with gastrointestinal cancer is significantly lower than that in healthy controls (Li et al., 2022b). Moreover, higher levels of *Bl. producta* were observed in the gut microbiota of UC patients who recovered following fecal microbiota transplantation (FMT) (Toscano et al., 2017). Recent research indicates that butyrate produced by *Bl. producta* exerts an inhibitory effect on neuroinflammation by modulating the RAS-NF-κB signaling pathway (Liu et al., 2024). In our study, we demonstrated for the first time that *Bl. producta* effectively improves colitis, highlighting its potential as a promising target for the discovery of new prebiotics. Additionally, our findings indicate that *Bl. producta* plays a crucial role in the anti-UC effects of various traditional Chinese medicines through modulation of the gut microbiota. This provides a novel perspective on how traditional Chinese medicines may improve disease outcomes via gut microbiota interactions.

However, several limitations must be addressed to validate these findings further. First, while antibiotic pre-treatment in the BP group introduced differences in baseline microbiota compared to the Model group, this design specifically aimed to isolate the therapeutic effect of *Blautia producta* in a microbiota-depleted environment. Future studies incorporating an antibiotic-only control group would further clarify the role of endogenous microbiota in DSS-induced colitis. Second, the strain of *Bl. producta* used to treat colitis in mice was derived from human feces. To more accurately assess its anti-colitis effects, a specific strain of *Bl. producta* should be isolated from mice subjected to HL/HB. Third, while the initial model employed was the large intestine damp heat model, the model used to verify the efficacy of the strain was the DSS-induced UC model. Although these models share certain similarities, they are not entirely congruent. Finally, the molecular mechanism of *Bl. producta*’s anti-colitis effect still needs further exploration.

## 5 Conclusion

In conclusion, in this study, we have successfully established a colitis model that is more in line with the TCM large intestine damp-heat syndrome (LIDHS). HL and HB, but not HQ, are effective in mitigating LIDHS colitis. Furthermore, the gut microbiota, especially the increase of *Blautia* and *Akkermansia*, is closely related to HL/HB’s therapeutic effects on LIDHS colitis.

## Data Availability

The data presented in the study are deposited in the NCBI repository, accession number PRJNA1273317.

## References

[B1] BeníTEZ-PáEZ A. GóMEZ Del Pugar E. M. LóPEZ-Almela I. Moya-PéREZ Á. CodoñER-Franch P. Sanz Y. (2020). Depletion of Blautia species in the microbiota of obese children relates to intestinal inflammation and metabolic phenotype worsening. mSystems 5, e00857. 10.1128/mSystems.00857-19 32209719 PMC7093825

[B2] Bolte L. A. Vich Vila A. Imhann F. Collij V. Gacesa R. Peters V. (2021). Long-term dietary patterns are associated with pro-inflammatory and anti-inflammatory features of the gut microbiome. Gut 70, 1287–1298. 10.1136/gutjnl-2020-322670 33811041 PMC8223641

[B3] Cai Y. Li X. Han Q. Bai J. Zheng Q. Sun R. (2023). Si-Ni-San improves experimental colitis by favoring Akkermensia colonization. J. Ethnopharmacol. 305, 116067. 10.1016/j.jep.2022.116067 36586523

[B4] Chen B. Dong X. Zhang J. L. Sun X. Zhou L. Zhao K. (2024). Natural compounds target programmed cell death (PCD) signaling mechanism to treat ulcerative colitis: a review. Front. Pharmacol. 15, 1333657. 10.3389/fphar.2024.1333657 38405669 PMC10885814

[B5] Cui L. Guan X. Ding W. Luo Y. Wang W. Bu W. (2021). Scutellaria baicalensis Georgi polysaccharide ameliorates DSS-induced ulcerative colitis by improving intestinal barrier function and modulating gut microbiota. Int. J. Biol. Macromol. 166, 1035–1045. 10.1016/j.ijbiomac.2020.10.259 33157130

[B6] Earley H. Lennon G. Balfe Á. Coffey J. C. Winter D. C. O'Connell P. R. (2019). The abundance of Akkermansia muciniphila and its relationship with sulphated colonic mucins in health and ulcerative colitis. Sci. Rep. 9, 15683. 10.1038/s41598-019-51878-3 31666581 PMC6821857

[B7] Guo X. Y. Liu X. J. Hao J. Y. (2020). Gut microbiota in ulcerative colitis: insights on pathogenesis and treatment. J. Dig. Dis. 21, 147–159. 10.1111/1751-2980.12849 32040250

[B8] Jang J. Y. Im E. Kim N. D. (2023). Therapeutic potential of bioactive components from Scutellaria baicalensis Georgi in inflammatory bowel disease and colorectal cancer: a review. Int. J. Mol. Sci. 24, 1954. 10.3390/ijms24031954 36768278 PMC9916177

[B9] Jiang L. Zhang J. Zhu B. Bao X. Tian J. Li Y. (2025). The aqueous extract of Reynoutria japonica ameliorates damp-heat ulcerative colitis in mice by modulating gut microbiota and metabolism. J. Ethnopharmacol. 338, 119042. 10.1016/j.jep.2024.119042 39515678

[B10] Le Berre C. Honap S. Peyrin-Biroulet L. (2023a). Ulcerative colitis. Lancet 402, 571–584. 10.1016/S0140-6736(23)00966-2 37573077

[B11] Li M. X. Li M. Y. Lei J. X. Wu Y. Z. Li Z. H. Chen L. M. (2022a). Huangqin decoction ameliorates DSS-induced ulcerative colitis: role of gut microbiota and amino acid metabolism, mTOR pathway and intestinal epithelial barrier. Phytomedicine 100, 154052. 10.1016/j.phymed.2022.154052 35344714

[B12] Li M. Y. Wu Y. Z. Qiu J. G. Lei J. X. Li M. X. Xu N. (2023). Huangqin Decoction ameliorates ulcerative colitis by regulating fatty acid metabolism to mediate macrophage polarization via activating FFAR4-AMPK-PPARα pathway. J. Ethnopharmacol. 311, 116430. 10.1016/j.jep.2023.116430 36997133

[B13] Li H. Li H. Stanton C. Ross R. P. Zhao J. Chen W. (2024). Exopolysaccharides produced by Bifidobacterium longum subsp. longum YS108R ameliorates DSS-induced ulcerative colitis in mice by improving the gut barrier and regulating the gut microbiota. J. Agric. Food Chem. 72, 7055–7073. 10.1021/acs.jafc.3c06421 38520351

[B14] Li J. Yang S. Lei R. Gu W. Qin Y. Ma S. (2018). Oral administration of rutile and anatase TiO2 nanoparticles shifts mouse gut microbiota structure. Nanoscale 10, 7736–7745. 10.1039/c8nr00386f 29658026

[B15] Li N. Bai C. Zhao L. Ge Y. Li X. (2022b). Characterization of the fecal microbiota in gastrointestinal cancer patients and healthy people. Clin. Transl. Oncol. 24, 1134–1147. 10.1007/s12094-021-02754-y 35167015

[B16] Liu J. Lv X. Ye T. Zhao M. Chen Z. Zhang Y. (2024). Microbiota-microglia crosstalk between Blautia producta and neuroinflammation of Parkinson's disease: a bench-to-bedside translational approach. Brain Behav. Immun. 117, 270–282. 10.1016/j.bbi.2024.01.010 38211635

[B17] Li Y. Shi X. Zhang J. Zhang X. Martin R. C. (2014). Hepatic protection and anticancer activity of curcuma: a potential chemopreventive strategy against hepatocellular carcinoma. Int. J. Oncol. 44, 505–513. 10.3892/ijo.2013.2184 24270742 PMC3898719

[B18] Pathmanathan S. G. Lawley B. Mcconnell M. Baird M. A. Tannock G. W. (2020). Gut bacteria characteristic of the infant microbiota down-regulate inflammatory transcriptional responses in HT-29 cells. Anaerobe 61, 102112. 10.1016/j.anaerobe.2019.102112 31629806

[B19] Qi J. Pan Z. Wang X. Zhang N. He G. Jiang X. (2024). Research advances of Zanthoxylum bungeanum Maxim. polyphenols in inflammatory diseases. Front. Immunol. 15, 1305886. 10.3389/fimmu.2024.1305886 38343532 PMC10853423

[B20] Santana P. T. Rosas S. L. B. Ribeiro B. E. Marinho Y. De Souza H. S. P. (2022). Dysbiosis in inflammatory bowel disease: pathogenic role and potential therapeutic targets. Int. J. Mol. Sci. 23, 3464. 10.3390/ijms23073464 35408838 PMC8998182

[B21] Schierová D. Březina J. MráZEK J. Fliegerová K. O. Kvasnová S. Bajer L. (2020). Gut microbiome changes in patients with active left-sided ulcerative colitis after fecal microbiome transplantation and topical 5-aminosalicylic acid therapy. Cells 9, 2283. 10.3390/cells9102283 33066233 PMC7602113

[B22] Song J. W. Long J. Y. Xie L. Zhang L. L. Xie Q. X. Chen H. J. (2020). Applications, phytochemistry, pharmacological effects, pharmacokinetics, toxicity of Scutellaria baicalensis Georgi. and its probably potential therapeutic effects on COVID-19: a review. Chin. Med. 15, 102. 10.1186/s13020-020-00384-0 32994803 PMC7517065

[B23] Sosna B. Aebisher D. Myśliwiec A. Dynarowicz K. Bartusik-Aebisher D. Oleś P. (2023). Selected cytokines and metalloproteinases in inflammatory bowel disease. Int. J. Mol. Sci. 25, 202. 10.3390/ijms25010202 38203373 PMC10779120

[B24] Sun Y. Zhong S. Yu J. Zhu J. Ji D. Hu G. (2018). The aqueous extract of Phellinus igniarius (SH) ameliorates dextran sodium sulfate-induced colitis in C57BL/6 mice. PLoS One 13, e0205007. 10.1371/journal.pone.0205007 30289941 PMC6173430

[B25] Su S. Wang X. XI X. Zhu L. Chen Q. Zhang H. (2021). Phellodendrine promotes autophagy by regulating the AMPK/mTOR pathway and treats ulcerative colitis. J. Cell. Mol. Med. 25, 5707–5720. 10.1111/jcmm.16587 34002930 PMC8184668

[B26] Toscano M. De Grandi R. Stronati L. De Vecchi E. Drago L. (2017). Effect of Lactobacillus rhamnosus HN001 and Bifidobacterium longum BB536 on the healthy gut microbiota composition at phyla and species level: a preliminary study. World J. Gastroenterol. 23, 2696–2704. 10.3748/wjg.v23.i15.2696 28487606 PMC5403748

[B27] Vesci L. Tundo G. Soldi S. Galletti S. Stoppoloni D. Bernardini R. (2024). A novel Lactobacillus brevis fermented with a vegetable substrate (AL0035) counteracts TNBS-induced colitis by modulating the gut microbiota composition and intestinal barrier. Nutrients 16, 937. 10.3390/nu16070937 38612971 PMC11013894

[B28] Wang G. Fan Y. Zhang G. Cai S. Ma Y. Yang L. (2024). Microbiota-derived indoles alleviate intestinal inflammation and modulate microbiome by microbial cross-feeding. Microbiome 12, 59. 10.1186/s40168-024-01750-y 38504383 PMC10949743

[B29] Wang H. Sun Y. Xiao F. J. Zhao X. Zhang W. Y. Xia Y. J. (2023). Mesenchymal stem cells ameliorate DSS-induced experimental colitis by modulating the gut microbiota and MUC-1 pathway. J. Inflamm. Res. 16, 2023–2039. 10.2147/JIR.S402592 37197438 PMC10184855

[B30] Wang J. Zhang C. Guo C. Li X. (2019). Chitosan ameliorates DSS-induced ulcerative colitis mice by enhancing intestinal barrier function and improving microflora. Int. J. Mol. Sci. 20, 5751. 10.3390/ijms20225751 31731793 PMC6888260

[B31] Wang Y. Cai Y. Li F. Zhang M. Wu Y. Dai Y. (2022). Effects of Scutellaria baicalensis Georgi. on intestinal flora in rats with spleen deficiency and damp-heat. J. Pharm. Biomed. Anal. 217, 114831. 10.1016/j.jpba.2022.114831 35609509

[B32] Wei Z.-J. Dong H.-B. Ren Y.-T. Jiang B. (2022). Efficacy and safety of fecal microbiota transplantation for the induction of remission in active ulcerative colitis: a systematic review and meta-analysis of randomized controlled trials. Ann. Transl. Med. 10, 802. 10.21037/atm-22-3236 35965832 PMC9372650

[B33] Wu Y. Ran L. Yang Y. Gao X. Peng M. Liu S. (2023). Deferasirox alleviates DSS-induced ulcerative colitis in mice by inhibiting ferroptosis and improving intestinal microbiota. Life Sci. 314, 121312. 10.1016/j.lfs.2022.121312 36563842

[B34] Xie Q. Li H. Ma R. Ren M. Li Y. Li J. (2022). Effect of Coptis chinensis franch and Magnolia officinalis on intestinal flora and intestinal barrier in a TNBS-induced ulcerative colitis rats model. Phytomedicine 97, 153927. 10.1016/j.phymed.2022.153927 35030387

[B35] Xue L. Zhao Y. Wang H. Li Z. Wu T. Liu R. (2023). The effects of live and pasteurized Akkermansia muciniphila on DSS-induced ulcerative colitis, gut microbiota, and metabolomics in mice. Food Funct. 14, 4632–4646. 10.1039/d2fo03493j 37098829

[B36] Xu W. Yu J. Yang Y. Li Z. Zhang Y. Zhang F. (2023). Strain-level screening of human gut microbes identifies Blautia producta as a new anti-hyperlipidemic probiotic. Gut Microbes 15, 2228045. 10.1080/19490976.2023.2228045 37408362 PMC10324434

[B37] Yanan Y. Xiaohui Z. Linen Z. Weiying L. Xiaopo Z. Chongming W. (2023). An analysis of gut bacterial and fungal community interactions in *Saxifraga stolonifera* curt.-treated mice. Dis. and Res. 3, 65–73. 10.54457/dr.202302003

[B38] Yang Y.-N. Deng Y.-T. Zang C.-C. Zhang F. Huang Z.-B. Dong L. (2022a). The gut microbial Co-abundance gene groups (CAGs) differentially respond to the flavor (Yao-Wei) of Chinese Materia Medica. Am. J. Chin. Med. 50, 2223–2244. 10.1142/S0192415X22500963 36266753

[B39] Yang Y. N. Wang Q. C. Xu W. Yu J. Zhang H. Wu C. (2022b). The berberine-enriched gut commensal Blautia producta ameliorates high-fat diet (HFD)-induced hyperlipidemia and stimulates liver LDLR expression. Biomed. Pharmacother. 155, 113749. 10.1016/j.biopha.2022.113749 36174380

[B40] Yao W. Yang C. Wen Y. Zhang W. Zhang X. Ma Q. (2017). Treatment effects and mechanisms of Yujin Powder on rat model of large intestine dampness-heat syndrome. J. Ethnopharmacol. 202, 265–280. 10.1016/j.jep.2017.03.030 28330724

[B41] Yao Y. Liu Y. Xu Q. Mao L. (2024). Short chain fatty acids: essential weapons of traditional medicine in treating inflammatory bowel disease. Molecules 29, 379. 10.3390/molecules29020379 38257292 PMC10818876

[B42] Yu W. Su X. Chen W. Tian X. Zhang K. Guo G. (2019). Three types of gut bacteria collaborating to improve Kui Jie'an enema treat DSS-induced colitis in mice. Biomed. Pharmacother. 113, 108751. 10.1016/j.biopha.2019.108751 30870717

[B43] Zhang B. Liu K. Yang H. Jin Z. Ding Q. Zhao L. (2022). Gut microbiota: the potential key target of TCM's therapeutic effect of treating different diseases using the same method-UC and T2DM as examples. Front. Cell. Infect. Microbiol. 12, 855075. 10.3389/fcimb.2022.855075 35433500 PMC9005880

[B44] Zhang L. Miao C. Wang Z. Guan X. Ma Y. Song J. (2024a). Preparation and characterisation of baicalin magnesium and its protective effect in ulcerative colitis via gut microbiota-bile acid axis modulation. Phytomedicine 126, 155416. 10.1016/j.phymed.2024.155416 38394726

[B45] Zhang Y. Han L. Dong J. Yuan Z. Yao W. Ji P. (2024b). Shaoyao decoction improves damp-heat colitis by activating the AHR/IL-22/STAT3 pathway through tryptophan metabolism driven by gut microbiota. J. Ethnopharmacol. 326, 117874. 10.1016/j.jep.2024.117874 38342152

[B46] Zhan J. Cheng J. Chang W. Su Y. Yue X. Wu C. (2025). Absolute quantitative metagenomic analysis provides more accurate insights for the anti-colitis effect of berberine via modulation of gut microbiota. Biomolecules 15, 400. 10.3390/biom15030400 40149936 PMC11940175

[B47] Zheng M. Han R. Yuan Y. Xing Y. Zhang W. Sun Z. (2022). The role of Akkermansia muciniphila in inflammatory bowel disease: current knowledge and perspectives. Front. Immunol. 13, 1089600. 10.3389/fimmu.2022.1089600 36685588 PMC9853388

[B48] Zhong S. Sun Y. Q. Huo J. X. Xu W. Y. Yang Y. N. Yang J. B. (2024). The gut microbiota-aromatic hydrocarbon receptor (AhR) axis mediates the anticolitic effect of polyphenol-rich extracts from Sanghuangporus. Imeta 3, e180. 10.1002/imt2.180 38882491 PMC11170970

[B49] Zou L.-E. Yang Y.-N. Zhan J. Cheng J. Fu Y. Cao Y. (2024). Gut microbiota-based discovery of Houttuyniae Herba as a novel prebiotic of Bacteroides thetaiotaomicron with anti-colitis activity. Biomed. and Pharmacother. 172, 116302. 10.1016/j.biopha.2024.116302 38387133

